# Integrating Deliberate Practice Into Group Supervision: A Case Illustration

**DOI:** 10.1002/jclp.23780

**Published:** 2025-02-25

**Authors:** Elisabet Rosén

**Affiliations:** ^1^ Institutt for Psykologi, Handelshøgskolen Innlandet—Fakultet for økonomi og samfunnsvitenskap Lillehammer Norway; ^2^ Department of Health, Education and Technology Luleå University of Technology Luleå Sweden; ^3^ Elisabet Rosén, ER dans & psykologi AB Umeå Sweden

**Keywords:** case illustration, deliberate practice, group supervision, psychiatric training, psychotherapeutic skills

## Abstract

One of many challenges in group supervision is the question of how to keep every participant engaged and fully available for learning. This paper describes a group supervision structure that uses Deliberate Practice (DP) to involve every participant in the group through the development of therapeutic skills intended to enhance the effectiveness of their work with clients. A case example illustrates how this process unfolded in the context of therapy with a specific client, conducted by a psychiatrist undergoing psychotherapy training in a deliberate practice supervision group. Analysis of video recordings of group supervision sessions and notes on solitary deliberate practice are used to illustrate how a DP structure engaged all participants in the group, while also facilitating the clinical development of the supervised psychiatrist and enhancing patient outcomes. A DP approach to group supervision enables participants to learn from each other's cases, through the deployment of flexible activities that engage all members of the group while also providing personalized skills training.

## 1 Introduction

Deliberate Practice (DP) is a learning methodology first devised by K. Anders Ericsson and colleagues, supported by an extensive programme of research into the development of expertize in high‐performing individuals in a range of professions (Ericsson et al. 1993). DP relies on purposeful repetitive practice of skills just beyond the practitioner's current capacity. A DP perspective emphasizes the importance of structure in training, including monitoring of the trainee's performance, clearly defined goals, repeated individual training on a delimited task, and guidance from a coach who progressively provides corrective feedback and adjusts the task as the trainee develops (Miller et al. 2023). In recent years, there has been a growing interest on how DP might be applied to the development of psychotherapeutic skills (Rousmaniere 2025; Rousmaniere et al. 2017; Rousmaniere and Vaz 2024; Husby 2023). This work has focused on basic therapist skills, such as alliance‐building, empathic responsiveness, and identifying and repairing alliance ruptures, skills that have been shown to have a significant impact on treatment outcomes (Wampold and Imel 2015), as well as the application of DP training principles for specific therapy models (e.g., Goldman et al. 2021).

Studies of DP further indicate that this learning method can be more effective than traditional didactic methods (Larsson et al. 2023; Westra et al. 2021). For instance, the first empirical study of DP suggested that the more time a therapist devotes to DP, the better their overall clinical effectiveness (Chow et al. 2015), regardless of educational level, gender, age, or years in the profession. Reviews by Mahon (2023) and Nurse et al. (2025) found consistent evidence for the effectiveness of DP for developing therapist skills across a number of studies.

### 1.1 Enhancing the Effectiveness of Clinical Supervision by Incorporating Deliberate Practice

DP emphasizes the importance of tailoring the training to each individual, to ensure the level of challenge of what is being practiced is optimal: not too hard, nor too easy. This individualized approach has influenced the use of DP within the field of psychotherapy supervision, through work that has focused on its application in formats where one supervisee is being trained by one DP supervisor, possibly with one additional person as an observer (e.g., Bawks et al. 2023). Recently, a general structure for individual DP supervision, the Sentio Supervision Model, has been developed by Rousmaniere and Vaz 2024 (Sentio Marriage and Family Therapy Program 2024). This flexible step‐by‐step protocol is designed to integrate deliberate practice into traditional supervision.

**Sentio Deliberate Practice Supervision Model**

1. **Opening of supervision:** Check on previously assigned DP Homework, if any.
2. **Ask supervisee to share and read the Supervision Preparation Form**
3. **Identify a clinical challenge:** What the client is saying/doing i.e. a challenge.
4. **Identify a skill deficit**: What the therapist tried doing in session that didn't work.
5. **Identify a small learning goal**: A new clinical skill to address the identified clinical challenge and skill deficit. This learning goal should be chunked down into actionable “skill criteria” to guide the next step of behavioral rehearsal.
6. **Behavior rehearsal (BR)**
  a. Repetitive rehearsal of identified learning goal/skill criteria.
  b. Supervisor or peer feedback (specific: positive and corrective).
  c. Assessing rehearsal difficulty, and adjusting if necessary.

7. **Closing Supervision**
  a. Assign DP Homework.
  b. Supervisor asks for feedback (e.g. what felt helpful/not so helpful?).

Following this DP supervision structure, one rather quickly works one's way through the initial steps: opening the session and reading through a preparation form used by the supervisee to identify what they have experienced as challenging with a specific client. The form includes a brief description of the patient's current problem, therapy goal, clinical challenge, and skill deficit (per the Sentio Supervision Model), identified by the supervisee, along with a 1–3 min video clip reference showing the issue. Ideally, the supervisor and supervisee then watch that video segment. If the supervisee struggles to select a video segment, the supervisor can help identify the most pressing skill deficit to address. Together they then formulate a small learning goal to address the identified clinical challenge. It is at this juncture that the heart of the skills training takes place: the phase of Behavioral Rehearsal. The intention behind this framework is to maximize the practice of skills that will have a tangible impact on the effectiveness of therapy with a specific client (Rousmaniere and Vaz 2024; Vaz and Rousmaniere 2022).

### 1.2 Deliberate Practice in a Group Supervision Context

In many educational settings and workplaces, it is common for clinical supervision to be conducted within a small group (Hawkins and McMahon 2020). The question of how to conduct DP supervision in a group setting raises issues around how DP can be individualized within a group while still keeping all members of the group engaged in a meaningful learning process (Axelsson et al. 2024; McLeod 2022; Rosén 2019). Working in a group creates a situation in which the whole is something besides the sum of its parts. Already early on Bion (1961) highlighted the possibility that interaction among group members can create both constructive and destructive effects on the group as a whole, because participation in a group may readily activate early maladaptive patterns of relating. Thus, while a group can provide a safe space for creative exploration together, it can also trigger destructively competitive dynamics that may jeopardize the work. For this reason, various forms of group supervision have been developed, that aim to foster the positive potential of a group while mitigating destructive group processes (Hawkins and McMahon 2020). Ögren and Boëthius (2012) suggested that it can be valuable to engage the group's resources and creativity by inviting reflection and roleplaying in a more or less structured manner. In this approach, the group participants engage in each other's supervision as well as being offered opportunities for developmental learning that focuses on their individual needs. Regular evaluation and monitoring of the work are further recommended, to ensure the group's safety and curb destructive group processes. The DP approach to group supervision described in the present article is informed by these principles. It adheres to the same sequence as the Sentio Supervision Model (used for one‐on‐one DP supervision), with a strong emphasis on the behavioral rehearsal (BR) phase. Importantly, the case study shows how group members are not mere bystanders or passive observers during the BR phase. Rather, they make an active contribution to learning as role‐play partners and providers of structured feedback to each other. The DP protocol is a supervisor‐led, task‐focused group format that promotes safety and minimizes the risk of the group shifting into a therapy group, a common challenge in process‐oriented supervision (Proctor 2008). It also provides a structured framework to help the supervisor navigate interpersonal dynamics within the group.

### 1.3 Behavior Rehearsal in DP Supervision

The behavioral rehearsal (BR) component of DP typically consists of short role‐plays based on an isolated patient statement, followed by a therapist‐improvised response, then feedback, difficulty assessment, adjustment, and repetition. In individual DP supervision, BR is ideally anchored in video‐recorded segments of therapy: the supervisee plays the video, and pauses it after the patient presents with a challenging statement/scenario. The supervisee then tries out different responses according to an agreed learning goal (e.g., empathic reflection), with specified skill criteria (e.g. reflect using the patient's words; use a tentative tone of voice) to improve therapeutic skills. BR can also be conducted through simulations where the supervisor takes the role of the patient, using the same challenging prompt from the video segment, and letting the supervisee try out responses in the role of therapist. Alternatively, the supervisor can step into the role of therapist, to model facilitative ways of responding to a patient, letting the supervisee to play their own patient, including imagining how the given response would be received by that patient.

The structure for using DP's BR in a group setting creates additional options. In a group, the BR phase can be personalized to the therapeutic challenge identified by a specific member, while the skills training will be generic for the others in the group at that moment. DP supervision in a group format gives participants the opportunity to observe varied responses to the same situation, potentially enhancing their therapeutic repertoire. A supervisee can, as in individual supervision, roleplay either therapist or patient and let another group member play the other role. A group setting also allows for further solutions, such as letting two supervisees roleplay a clinically challenging scenario or letting the supervisor, model the tricky situation in the role of therapist, with the supervisee in the role of their patient, letting the rest of the group members give feedback on how well the supervisor handled the skill criteria. The supervisor can preferably perform a roleplay that demonstrates poor skills, which gives the group members an opportunity to train giving accurate corrective feedback. This also helps balance power dynamics between supervisees and the supervisor, which can otherwise become skewed in task‐focused supervision (Proctor 2008).

The feedback that follows on BR is central in DP training, as a means of fostering the supervisee's development. Effective feedback is specific, actionable, positive and corrective. The DP supervisor maintains the DP structure by ensuring each group member provides balanced feedback on the agreed‐upon skill criteria for the roleplay. Supervisees are responsible for giving feedback on one skill criterion each, requiring all members to contribute in a structured way (e.g., one supervisee tracks and provides feedback on the colleague's ability to use a tentative tone of voice during rehearsal, another on how the use of patient's words are reflected). After the first roleplay, feedback is usually given before the next one. Later, several roleplays may be done consecutively to maintain training momentum. This approach gives supervisees ample opportunities to practice giving and receiving constructive feedback. The supervisor reinforces the value of feedback by inviting the group to reflect on their supervision experience in the end of each session, and in the preparation form, facilitating early detection of alliance ruptures within the group (Ögren and Boëthius 2012).

Following feedback, assessing the difficulty level of the skill being trained is key for optimal learning. This is done by asking the roleplayed “therapist” how challenging the BR task felt, and how hard it was to fulfill skill criteria during rehearsal. The therapist evaluates this on a scale from 0 to 10, based on their perceived bodily sensations, impulses, feelings and thoughts, aiming for a difficulty level between 3 and 7, for optimal learning (Rousmaniere 2025). The supervisor can also assess difficulty by observing non‐verbal cues: a supervisee appearing too relaxed (too easy), sighing or squirming (good challenge), or becoming numb or blank (too hard). Adjustments are made accordingly, before inviting for further training. The structured DP protocol provides safety for both group members and the supervisor, as participants take on multiple roles, and thus function as a “co‐supervisor.” The supervisor's role is to guide the group, prioritizing active learning by BR practice, while minimizing passive psychoeducation or extensive theoretical discussions. These topics are instead referred to the literature and lectures outside the scope of DP supervision.

At the end of supervision, everyone receives homework based on the session's work. This may also include inner skill exercises (Therapist Self‐Awareness; Bawks et al. 2023; Goldman et al. 2021; Section 2.3.1) to help supervisees privately process their reactions to the therapeutic material. In this way DP addresses transferences and countertransferences through practice rather than conceptual discussion, making it especially suitable for groups while reducing the risk of uncomfortable exposure, a more common issue in process‐oriented group formats (Proctor 2008).

Between supervisions, supervisees reflect on their homework using a DP Therapist Diary Form (Rousmaniere 2025). Here they document the task, how it was completed (alone or with a colleague), what they found helpful and unhelpful, and what they learned about themselves and wish to explore further. They are reminded to share only what they feel comfortable disclosing, as the reflections are shared with the group before the next supervision.

## 2 Case Illustration

The following case illustration describes how the behavioral rehearsal (BR) phase of DP is structured in supervision groups within a psychotherapy training programme for psychiatrists in Sweden. All four supervisees in the group, as well as the patient of one of them, gave consent for their information to be used for this case illustration. To protect their identities, all names have been changed and some details have been omitted.

The supervision group consisted of psychiatrists receiving basic psychotherapy training as part of their postgraduate speciality training, regulated by the Swedish National Board of Health and Welfare. The essence of this training, is to learn the fundamental psychotherapeutic tools, including both common factors and method‐specific techniques, in theory and practice. Since 2007/2008, METIS ‐ The Swedish Psychiatric Residency education project, has been responsible for developing psychiatric curricula, including psychotherapy. The METIS psychotherapy course in Northern Sweden spans over three terms, with eight 3‐day theoretical course meetings, personal tutorials, recommended reading and homework (for the therapeutic work, presented under 2.3). Topics included in the curriculum are Communicative Competency, Developmental Psychology, Psychotherapeutic Theory and Practice, Diagnostics, Evaluation, Research, Combining Medical and Psychological Treatment, and conducting Psychotherapy under supervision. Alongside course meetings, participants are required to conduct psychotherapy with two patients on a weekly basis. The patients are recruited from regional psychiatric clinics. Most, including the patient in this case illustration, have undergone prior psychiatric interventions. Course members receive psychotherapeutic supervision every other week for 1 year, mostly online. The course aims to enhance psychiatrists' interpersonal communication skills through action‐based learning and practical exercises, using DP as a key learning tool. All therapy sessions were video recorded, and selected segments were used in the DP supervision. They are given an orientation in different evidence‐based psychotherapy methods (e.g. cognitive behavior psychotherapy, CBT; psychodynamic Affect Phobia Therapy, APT; McCullough et al. 2003), with focus on common factors (Wampold and Imel 2015). The overall objective of the training is to develop basic psychotherapeutic skills, that participants can use in their work as psychiatrists. This METIS course also includes action‐based DP workshops for the clinical supervisors each term, to reinforce basic principles of DP and review strategies for handling alliance ruptures in the supervision groups.

Psychiatrists frequently encounter patients with severe problems, requiring them to make quick decisions and shoulder significant responsibilities. Often, they do not know if they will see the same patient again. They must assess, diagnose, and provide a clear treatment plan, which may include medication or hospitalization, ideally in a single meeting. They work irregular hours, adapting their schedules as needed for emergencies. Their profession commands high status in society, making them accustomed to both respect and scrutiny. Supervisors must take these unique factors into account when planning and conducting supervision for this group. The challenge is for psychiatrists, patients, supervisors, and METIS to collaborate effectively for the benefit of the patient. A key focus of the programme is to enable participants to shift from a medical, prescriptive model, where a specific diagnosis is automatically matched with a particular treatment—to a more contextual model that is responsive to individual circumstances (Wampold and Imel 2015). Participants are supported to adopt a more collaborative, and equal stance towards the patient, that includes slowing down and listening to the patient's own understanding of their problems, rather than telling them and giving them instant recommendations, and trusting the capacity of the patient to heal and grow.

The supervision group consisted of four supervisees (one woman and three men): Ava, John, Liam and Teo. The overarching learning goal for DP group supervision was to help supervisees improve in basic psychotherapeutic skills, and in particular to help them to become more able to tolerate strong emotions. The core theoretical model underpinning the training—Affect Phobia Therapy (APT; McCullough et al. 2003) – has a strong focus on the therapeutic value for patients of knowing and being able to deal with their emotions, as a strategy for developing better relationships with oneself and others in one's life. Competence in delivery of APT requires supervisees to learn to accept their own feelings, through self‐awareness, as a prerequisite for being equipped to engage with their patients' strong emotions. The use of DP groupwork in this setting is illustrated through an account of the experience one of the supervisees in the group, and his work with one of his patients.

### 2.1 Presenting Problem and Client Description

The supervisee, Teo, was a 34‐year‐old male psychiatrist, with 4 years of psychiatric experience, working at a university hospital in Sweden. The supervisee's goal with this psychotherapy course was to become more competent and confident in engaging more openly with patients and enhance listening and collaboration for better patient care.

The patient, “Pia”, was a young, woman in her 20 s, diagnosed with emotionally unstable personality disorder (EUPD/Borderline Personality Disorder). Pia exhibited unstable relational patterns where she either loved or hated people, including herself. She reported experiencing either intense feelings or feeling flat and empty. She regularly engaged in destructive sexual behavior to either get herself to feel something or as a self‐punishing strategy. She had recurrent major depression and suffered from unstable self‐worth where she, on the one hand, felt that she deserved to have a good life, and on the other hand, saw herself as a bad person who deserved punishment. Her goal for the therapeutic treatment is to get to know her feelings, to stand up for herself. She wants to be able to handle her feelings in a better way, especially in relationship to herself and in other close relationships, to be able to create more stability in life.

### 2.2 Case Formulation

This closed supervision group comprised the four psychiatrists mentioned earlier. While all worked in psychiatry, none had prior psychotherapy training or knowledge of DP. Each met with two patients and could choose which patient to present during their 50 min supervision slot. No other patients were discussed. The group met on 27 occasions, for 4 h each time. The patient, presented here, received 27 sessions over a period of 1 year. From an Affect Phobia Therapy (APT) perspective, the supervisee Teo's supervision goal was to empathically identify his own skill deficits, in terms of affect phobias, affirming the disclosing of them in supervision as a basis for working through obstacles for being able to communicate emotions more openly and feeling safe enough to tolerate vulnerability, while collaborating in both supervision and therapy. The APT case formulation for Pia was centered around working through her affect phobias by empathically and affirmingly supporting her to become more aware of how her relational patterns were constricted by emotional avoidance. It was anticipated that such a process might help her be better able to empathize with herself, rather than being self‐critical, and thereby increasing her tolerance towards herself and others, and ultimately enhancing her capacity for self‐care. To assist the supervisees, they had to account for their thoughts on the patients' problematic pattern in the preparation form: identifying their triggering feelings and avoidance strategies, where these patterns appear in the patient's current life (including transferences with the therapist), and their historical origins ‐ a *Core Conflict Formulation* (McCullough et al. 2003, Chapter 4).

### 2.3 Course of Supervision and Treatment

The following sections track the course of supervision and treatment using segments from the early, middle, and final phases of group supervision. In addition, the presentation also explores how the supervisee, Teo, used DP to develop clinical skills and self‐awareness, and their corresponding impact on the treatment progress with the patient Pia. Each vignette begins with Teo's description of a clinical challenge, as written in his supervision preparation form, before moving on to identify the supervisee's own skill deficit, either spotted by Teo himself, or by the supervisor via watching a therapy video recording. From here, supervisor and supervisee collaboratively identify a learning goal with actionable skill criteria, which form the basis for the ensuing behavioral rehearsal (BR) work. Early reading recommendations included: What is an Affect Phobia?; Psychodynamic Conflict and Malan's Two Triangles; How to Assess the Patient and Select Treatment (McCullough et al. 2003, Chapter 1‐3).

#### 2.3.1 The Early Phase of Supervision and Therapy

Two examples of group‐based BR activities relating to the early phase of therapy are outlined below. The first occurred in a supervision session *prior* to the first meeting with the client and referred to skills associated with the very beginning of therapy. The second activity relates to challenges arising in the first session with the client.

- Clinical challenge: The therapist/supervisee, Teo, as well as the other three supervisees in the group, used extracts from the psychiatric files of their respective first assigned patients, to identify the considerable challenges associated with the task of moving from a psychiatric to a psychotherapeutic mode of relating with an individual with longstanding mental health difficulties.
- Skill Deficit: All four supervisees essentially asked the same question: *How do I start?*
- Learning Goal: How to start psychotherapy by empathically inviting the patient to share the problem(s) that they would hope to address in therapy, and identify a preliminary formulation of the patient's affect phobia pattern (McCullough et al. 2003, Chapter 4; Goldman et al. 2021, Exercise 2, Empathic Understanding).
- Skill being practised: Exploring the patient's problem and reasons for coming to therapy: welcoming and greeting the patient (establishing contact), inviting them to describe their problem, and asking for a concrete example.
- BR group practice: We practiced in a circle in which one participant started by taking the role as therapist, and another group member in the role as patient (as described in the therapist's earlier summary of case notes). This activity proceeded at a fairly rapid pace (not unlike speed‐dating) as a means of maximizing involvement, maintaining energy levels, and allowing participants to experiment with a range of different possible responses: it starts with an interaction between therapist and patient, and then roles are switched as each group member turns to the next person in the circle. This way the supervisees do not stay long in focus at a time, and therefore it is not so anxiety‐provoking for the supervisees. They have the same task and can be inspired by each other's way of phrasing it.

The supervisor started by modeling in the role as therapist, greeted Teo (who played the role of the patient) and asked about their problems. Teo answered with a statement taken from Pia's case notes: “*I feel depressed and destroy things for myself when I get so angry all the time”*. The supervisor then asked about a specific example. At this point, this short roleplay ended and the roles were switched: Teo, now in the role of therapist, greeted another supervisee in the group, Liam, who now played the patient, and practiced asking for a specific example. This same roleplaying process was rotated to that all members of the group had a chance to practice: Liam then greeted and asked Ava, and Ava greeted and asked John, and John greeted and asked the supervisor. All this is considered one round of behavioral rehearsal. We then assessed the difficulty of the task, adjusted it where needed, gave feedback on everyone's use of the skill, and repeated this brief roleplaying activity again over several rounds.

At the next supervision session, Teo reported that, on their first therapy session, Pia had described her goal for therapy as: “*I want to be able to handle my emotions in a balanced way and to like myself*. Teo added that: “*She's shut down and let herself be walked over. She wants to stand up for herself, but is afraid to do so”*. Following the reading of his Supervision Preparation Form, Teo described the clinical challenges that had emerged for him around beginning Pia's treatment. He recognized that he had been too eager to quickly move ahead in the treatment, and had a hard time resisting telling her what to do, rather than slowing down and asking her instead. He felt uncomfortable when Pia expressed strong emotions, including thoughts and feelings around not wanting to live. Teo identified his main skills deficit (i.e., what he did in session that didn't work) as: “*I'm changing the subject: I keep suggesting that we talk about something else”*. Together, we identified the need to help him develop more effective skills around taking a not‐knowing stance, slowing down, staying with emotions, and reflecting back rather than quickly try to solve the client's problems. During session when Pia asked herself “*why can't I accept help?”*, Teo then reflected in supervision: “*I tend not to dig deeper”*. In supervision, we worked on the psychotherapeutic importance of exploring a Pia's relational patterns and validating that they are understandable due to her background, where no one cared before, and affirm that she now has the courage to reveal this in therapy, even though she's afraid to accept help.

More concretely, the BR used to address Teo's skills deficit, which was similar to those presented by other members of the supervision group, was to work on empathic reflection (Goldman et al. 2021) in response to patient statements that had elicited a problem‐solving response from the therapist. The aim of this DP activity was to develop Teo's capacity to tolerate strong feelings, collaborate with the patient to a greater extent, and eventually explore Pia's relational patterns. Ultimately, this practice might help the patient take some steps toward developing a better understanding of her relational patterns, primarily around not trusting people because nobody in her early life seemed to care. These shifts might mean that in therapy she could now start to relate in a different way: she could ask for, and receive help.

Homework here was on the inner skill, therapist self‐awareness: reviewing the same video segment, while observing inner reactions (countertransferences), trying to stay with strong feelings, and notice bodily sensations, feelings thoughts and impulses to act (Bawks et al. 2023; Goldman et al. 2021; McCullough et al. 2003, Chapter 7).

#### 2.3.2 The Middle Phase of Supervision and Therapy

In this section, examples of how DP assisted Teo to make progress in his work with Pia, are briefly explored in terms of the tasks and phases specified by the supervision model. Each member of the supervision group was also engaged in this process and began to present personalized challenges in response to the ongoing group skills training.

*Responding constructively to patient self‐criticism*

Teo took part in three separate DP supervision sessions to develop the skill on how to respond constructively to Pia's tendency to exhibit a harsh inner critic that undermined her capacity to develop close relationships. The first session comprised:

- Clinical challenge: Pia is very self‐critical and over‐adapts to make others like her.
- Therapist's Skill Deficit: Teo: “*What can I do when she becomes so self‐critical? I mostly just listened.”*
- Learning goal: How to replace the self‐attacking with more self‐affirming behavior (McCullough et al. 2003, Chapter 5, 6 and 9, Defense, and Self‐Care work).
- Behavioral Rehearsal: Skill criteria: (1) Highlight the patient's self‐criticism; (2) Invite the patient to reflect on the meaning of self‐criticism in the context of their life history. (3) Invite exploration to promote self‐care instead.

In the following supervision session, Teo reported that Pia had continued to criticize herself in their most recent therapy session. He played a video segment that began with Pia saying that men only want her for sex and that she feels that she is no good for anything else:

Pia (in video): Why do I even exist? What is the purpose?

Teo (in video): It might also make you criticize yourself?

Pia (in video): Yes!

Teo (in video): And that's something really important that you shouldn't do.

Pia (in video, visibly upset): I can't help it, I can't stop it. [*video stops*]

Teo (in supervision): I tried to stop her self‐criticism, as we trained. But she got upset and shut down. It went wrong. I'm not really sure how I should approach her in this situation? If I were in my doctor role, I would have distracted her. But now we're supposed to dive into the difficult stuff. Before, you've seen me helping her to avoid it…

Supervisor: It's not wrong to point out that being self‐critical isn't helpful. It's about how you do it, that might make her even more self‐critical for being self‐critical.

In accordance with the DP supervision model and to promote collaboration, at this point the supervisor starts by asking the supervisee for ideas on a learning goal to focus on for behavioral rehearsal.

Supervisor: When you watch this, is there anything you would like to do differently?

Teo: Hm… Maybe intervene earlier…

We agreed on an overarching learning goal of *empathic validation* (Bawks et al. 2023; McCullough et al. 2003, Chapter 6, Defense work) specifically consisting of the Skill Criteria: (1) Validate (that it can be hard not to be self‐critical, due to her upbringing of critical parents', and ex‐boyfriends' critical attitude towards her); (2) Provide a brief psychoeducation (that being self‐critical doesn't help her in the long run); (3) Invite to explore alternative ways of taking care of herself when faced with challenging situations.

In the Behavioral Rehearsal (BR) phase, Teo practiced following these skill criteria by responding to a segment of the therapy recording. The provision of feedback for each skill criterion was distributed among the two other members of the supervision group, John and Ava:

Supervisor: Which skill criterion would you like to focus on here, Ava and John: validation, psychoeducation, or inviting collaborative exploration?

John: I can take the psychoeducation on self‐criticism, as I am wondering myself how to say it in a good way without sounding accusatory.

Ava: I'll take the validation at the beginning.

Supervisor: Then I'll keep an extra eye on the invitation to explore together. Let's go!

Later in the supervision session, feedback was offered in respect of the skill criterion:

Supervisor: Ava and John, how do you think Teo managed the skill criteria in this practice here?

Ava: It flowed. When you use the patient's own words, it becomes an immediate validation.

John: It was short, and you were very present and sympathetic, but I felt a little lost, it didn't point in any direction for getting help.

Supervisor: I agree with both of you and for that last part, the invitation got a bit lost here. I think we should try again.

Later, the supervisor assesses the rehearsal's difficulty while promoting safe boundaries (Rousmaniere 2025):

Supervisor: How did that practice feel for you? And remember to only share what you feel comfortable sharing.

Teo: It feels much better! Self‐criticism isn't good, but it's such a big part of her, which made it so hard to work with. I need to practice more to find the words though.

Already this supervisee has realized the importance to practice on his own, to find the words that would feel natural for him.

At the end of this practice session, the supervisor suggested two homework assignments: understanding the concept of *empathic validation* through reading and reflection, and preparing for the next therapy session by playing the very beginning of the video recording of the most recent session, pretending it is the next session, and repairing the rupture (Bawks et al. 2023: Goldman et al. 2021) by: reminding Pia of what happened in the last therapy session, inviting her to share how that had felt for her, and taking his own share of the responsibility of what happened in the last session.

At the next supervision meeting, Teo reported back on his solitary practice: “*I practiced with the video clip, making slight variations each time until I found a soft, short, and concise way. By trying to shorten it each time, it became more succinct. With more repetitions, it flowed better. Recording myself and watching it helped me see where I could improve. I thought rather quickly after a few practices that I had found a good method, but I kept practicing a bit longer and found even more things to optimize. I learned… that one shouldn't be too quick to think they're done and that it's not worth practicing more.”* Teo reported that it had been valuable for him to evolve his own way of training, and had realized that repetition was the key to developing competence.

During Pia's next session, Teo apologized for his rather abrupt way of cutting off her self‐critical thinking. In response, Pia mentioned that she had spent time since the last session reflecting on her strong feelings and self‐critical thoughts, and expressed gratitude for Teo's persistence in supporting her to handle her feelings in a calmer way.

#### 2.3.3 The Ending Phase of Supervision and Therapy

Behavioral rehearsal within a DP group supervision context was also used to develop skills for overcoming challenges associated with the termination of therapy. Toward the end of therapy, Pia had found her own way to process complex thoughts and feelings by writing in a journal. Pia then declared that it might be time to explore the possibility of ending therapy.

New Clinical Challenge: In one of the last sessions of therapy, Pia reported: “*It's better now than ever before.”* She laughs because she thinks she's handled everything so well:

Teo (in session): It seems you've managed quite well.

Pia: I think so too [Pia laughs and Teo smiles broadly]. I really felt that, damn, I *have* been managing pretty well with everything…

Teo: Yes…

Pia: … and it feels kind of very calm inside me too.

Therapist's Skill Deficit: At this point, Teo does not know what to do when the patient feels well.

Teo (in supervision): What should I do when she's feeling good? I don't really know. She's doing well. What do I do then?

Learning goal: This might be a situation when psychiatrists would typically end treatment. However, in a psychotherapy setting it represents a golden opportunity to really anchor the change experienced by the patient, by reflecting on it or meta‐processing it. The supervisor therefore proposed the learning goal of *developing an ability to facilitate meta‐reflection*, on those good feelings and termination of therapy, (Bawks et al. 2023; McCullough et al. 2003).

Supervisor: How does that sound to you?

Teo: Yes, that sounds good. Like it's reinforcing those clinical gains even more.

Supervisor: Yes, exactly! Reinforce it, and give a small rationale for it, so she can recognize it and benefit from it when the therapy is over.

For this new learning goal and behavioral rehearsal, the supervisor proposed these Skill Criteria: (1) To meta‐reflect on the patient's feelings about the therapeutic work done together; (2) Give a rationale for how what has been learned in therapy might be maintained. This time around, there was only one more supervisee in the group from which to distribute the provision of feedback for these two skill criteria:

Supervisor: John, what would you like to focus on here: giving feedback for “1. Meta‐reflecting on the feeling”, or “2. Giving the rationale”?

John: I'll take the rationale.

Supervisor: Okay, I'll keep an eye on how Teo meta‐reflects on the feeling. Let's try it!

After some rounds of rehearsal, the supervisor assesses difficulty:

Supervisor: Ok, we'll stop there. How did that feel, Teo?

Teo: Yes, it felt OK, but maybe I could have been a bit clearer on some points.

Here the supervisor also stops the role‐play to make sure it doesn't become too long and moves to the feedback phase of rehearsal:

Supervisor: John, what did you think about Teo's provision of a rationale here?

John: I think overall it went well. Maybe you can shorten it a bit.

Supervisor: Yes, I agree, you covered all the parts, and a nice exploration on her feelings. I think we can polish it a bit, by shortening it as John says.

Teo tries it again and is successful in making his intervention much shorter.

Teo: I wanted to ramble on, but I stopped! It was short, but maybe that's better.

John: I thought it was very good! In a tenth of the time and words, it was the same thing.

Rehearsal difficulty assessment and adjustment phase:

Supervisor: Yes, indeed! Am I correct in thinking that this is not too hard on you Teo?

Teo: No, it's okay. We can make it harder.

Supervisor: Nice! I was thinking, it's not just about acknowledging the client's calmness. There's also laughter there. Maybe you could mention both, and ask her about it.

Teo: You're right. There's maybe also a bit of surprise on her end. Yes, let's try that.

After rehearsing an intervention that addresses both the patient's calmness and laughter:

Teo: Yeah, it feels good to bring up the laughter too. It captures the whole situation. I guess then it becomes even more enjoyable for her too.

John (providing feedback): I think it's good. She will find it easier to notice the different feelings herself then.

Another round of difficulty assessment and adjustment:

Supervisor: Yes. I just want to check: How difficult does this practice feel now?

Teo: It's not that difficult.

Supervisor: Okay. Now, have you seen how you yourself look, as she laughs?

Teo: No, but I laugh when she laughs.

Supervisor: Of course you do! You have done this work together, and if you want, you can tell her that her laughter also makes you happy for her. What do you think?

Teo: Yes, I can try. Let's go again!

Notice that, after a few rounds of rehearsal, the supervisor now adjusted for optimal learning and added a third skill criterion for the supervisee to practice: for the therapist to self‐disclose his own feelings about the client's positive changes and reactions (Goldman et al. 2021).

After a few rounds of rehearsing all three skill criteria, the group again provides feedback:

John: I think it was short and concise! Clear.

Supervisor: Nice!

Teo: Yes, it feels good. It's contagious, that we are all in the same boat, seeing the client's change, and it's incredible.

John: Yes, it's fun to see! And it's a big difference. She feels like a completely different person!

At this point, as the behavioral rehearsal phase is coming to an end, the supervisor takes the opportunity to remind and elaborate on the rationale for using this skill with this client. While this was touched on briefly at the beginning of practice, we have found that it is important that supervisors do not take up too much time in this sort of intellectual conceptualizing before any practice has occurred, since this can easily take away from precious rehearsal time. Having completed multiple rounds of rehearsal, the supervisor concludes:

Supervisor: The purpose of doing all this with her is in communicating that you've done this work *together*. You're communicating that she's not alone in this experience, and you are really happy for her. So, even your therapeutic self‐disclosure can have a reinforcing effect. And, by the way, it makes *me* happy as a supervisor to see her joy and your joy over what you have done together. Well done!

### 2.4 Outcome and Prognosis

In a supervision case study, it is necessary to consider two independent but hopefully interconnected outcome domains: the outcomes achieved by the supervisee (such as increased skills acquisition), and those for their patient (such as increased well‐being and decreased symptomatology).

#### 2.4.1 Outcome for the Supervised Psychiatrist Teo

Teo had been diligent in DP supervision, consistently bringing clear questions and relevant video clips from his work with Pia. He was eager to practice skills, and to support his colleagues in their own learning, observing that this benefitted his own skill development. He reported that seeing himself on film was the most beneficial aspect of DP: “*I needed to practice more to find the right words (…) but I'm not sure I agree that deliberate practice is essentially the only way to develop as a psychotherapist. I find that watching myself in the video recordings has been very beneficial, and helped me to identify my strengths and weaknesses.”* He also noticed that the DP supervision has changed him, both in his work and in his private relations: “*I feel that I sometimes might come across as if I have all the answers… I have been working on finding a better balance and being softer in tone, to help others feel safer and more open in conversations. I think in a slightly different way. It has been very rewarding and has helped me both personally, and in my work… My partner says that I've become kinder and listen better. That feels good*.” In addition to these specific psychotherapeutic skills, Teo also reported that DP had enabled him to conduct therapy in a more time‐efficient manner. He observed that his initial sessions with Pia had lasted up to 1.5 h, because it had taken a long time before the actual therapeutic work began during each session. After working on how to focus on a relevant patient issue and how to end a session, it became easier for Teo to finish on time. As a result, sessions became shorter and adhered to the agreed‐upon timeframe, while also seemingly becoming more therapeutically effective.

#### 2.4.2 Outcome for the Patient Pia

At the end of therapy, Pia reported that she still sometimes shut down, or became emotionally flat in her contact with others, but not to the same extent as before. She had been able to eliminate self‐sabotaging sexual behaviors and alcohol abuse. Instead, she observed that she now had a consistent sense of wanting to feel good and a much better understanding of how to work towards and maintain that state through structures and activities in life. Her safety, self‐worth, and self‐assertion have increased. However, she still occasionally felt anxiety that something bad would happen in life, causing her to momentarily lose the security and self‐worth she had been working towards. This makes her a little bit rigid about different activities, such as cleaning and exercising, to make sure to keep the good stuff going.

Objective outcome measurement was used via the Outcome Rating Scale (Miller and Bertolino 2012). This instrument showed that, over the course of therapy, Pia had improved her well‐being from within the clinical population range (score of 22.99), to within the nonclinical range (final score of 34.52). She associated this change with having been able to create new relational patterns where openness and repair in relationships were more possible for her. She noted that the experience of being able to repair an alliance rupture together with the therapist in therapy became a model for a healthy and balanced relationship, allowing mixed feelings to be tolerated, This aligned with her goals of increasing empathy for herself and others, strengthening self‐care, and letting go of self‐defeating defenses to adopt a more self‐compassionate stance (McCullough et al. 2003). At the end of therapy, Pia was confident that she was much better equipped to repairing future relational challenges in life.

## 3 Summary and Implications For Clinical Practice

The present case example illustrates some of the ways in which a DP‐informed group supervision can be adapted to meet the learning needs of supervisees. Because a DP format focuses on specific micro‐skills, it also proved necessary to supplement participant learning with theoretical readings that offered an overview of the therapeutic structure and process as a whole (McCullough et al. 2003). At later stages of the work in the group, supervisees were more able to be in the here‐and‐now both in supervision and treatment and to learn from each other. The frequently posed question by supervisees on “*what happens next?”* (after using a practiced skill) may be interpreted as reflecting the desire to quickly move forward, positioning the psychiatrist as the expert and the patient as a largely passive recipient of treatment. As a consequence, the members of this supervision group needed help to appreciate that true expertize in a psychotherapeutic context lies in genuinely listening to the patient and using the patient's language, thoughts, and ideas to collaboratively make the therapy effective, regardless of which therapy method one uses. The DP exercise *empathic validation* (Bawks et al. 2023; Goldman et al. 2021) really helped them with this skill. Such an approach to working with patients also eases the burden on psychiatrists to provide every solution themselves. It was a surprising experience for the psychiatrists in this group that the therapy relationship itself could be healing for the patient. These psychiatrists reported that they arrived at a point of recognizing that a conversation can be as effective as medication if only one can slow down, listen and use therapeutically what happens in the moment with the patient.

This case illustration also provides preliminary evidence for how the supervisee's capacity to use DP to facilitate competence gradually evolves over time, as participants learn how to apply it for their purposes. At the early stage of the group, supervisees felt more comfortable doing DP training by role‐playing with each other. In time, it became easier to also role‐play to video segments of their own patient, or flexibly mix these modes of skills rehearsal. Engaging everyone continuously in each other's training, by allocating role‐play and feedback tasks, made the group member under focus feel less exposed, and operated as a means of containing anxiety. This was particularly evident when training was carried out in role‐plays in a circle, or to video clips where each observer was responsible for providing balanced feedback on one skill criterion. The importance for the supervisor to be able to vary working strategies with each member in a group was a crucial factor in supporting learningEach Members of the group reported different types of challenges and needed different ways for staying in the learning zone and feeling safe. In this group, some were very clear about what they wanted help with, meanwhile, others took time to realize what to bring to supervision, preferring instead to show an “interesting” clip or discuss broad theory—activities that had the effect of reducing time for actual skills training.

Another notable evolution in this DP supervision group regards the provision of feedback during behavioral rehearsal. Initially, the feedback provided by, and between supervisees was mostly just positive and broad (e.g. “It sounded good to me”). Later on, feedback became more balanced and specific, and thus more actionable. Towards the end, they occasionally also began providing corrective feedback to the supervisor and even developed the ability to assist in maintaining the DP structure, for instance by offering encouraging comments to support the continuation of behavioral rehearsal of skills—A growth towards a more “cooperative—group‐led task group” (Proctor 2008). The learning is dual: They learned the basics of psychotherapy, and at the same time, they learned how to learn with the DPs strict structure, that still gives many creative possibilities in a group (McLeod 2022).

In their evaluation of the whole supervision experience, participants also consistently emphasized the importance of role‐playing the patient role as a means of becoming more sensitive to the significance of subtle nuances in the therapist's approach. The numerous roleplays likely enhanced their empathy for their patients, by increasing sensitivity to internal emotional experiences in themselves, addressing their own affect phobias (McCullough et al. 2003). This aligns with Teo's goals of overcoming obstacles to feeling safe enough to tolerate vulnerability. Compared to individual supervision, group work where participants engage in each other's cases offers more opportunities for regular practice in the patient role. The members' attitude towards DP is often ambivalent, in that it is challenging and effortful work, but also ultimately perceived as helpful in developing relevant skills to use with one's patients. The supervisees in this group identified the need for individual DP training between group supervision sessions, and at the same time, they found this to be the hardest part of DP. Introducing DP stepwise might be a good way to handle this ambivalence.

An interesting final observation was that analysis of video recordings of these supervision sessions revealed that the initial engagement of group participants during BR was lower than the supervisor had anticipated. This insight led the supervisor to distribute DP supervision tasks more extensively across participants, and the group participants also reported greater satisfaction with the work in supervision as a result. Even though the supervisor always has the responsibility for keeping the structure in DP supervision, different components of the DP format can gradually be delegated to the participants in the group, as they become more familiar with the DP structure. In this case illustration, participants engaged in the BR phase through skill‐building role‐plays and, as observers, in providing balanced feedback. Over time, the group improved in collaboratively formulating skill criteria and assessing and adjusting training difficulty, indicating a potential for further task delegation to enhance group engagement.

In conclusion, integrating DP into group supervision offers a dynamic, personalized framework for developing psychotherapeutic skills. The process fosters not only individual growth but also group collaboration, as supervisees learn through structured role‐plays, feedback, and iterative practice. By embracing the therapeutic relationship as a co‐created space for growth, participants shifted from a prescriptive approach to one that values listening and emotional attunement. This case study demonstrates how DP has the potential to enhance both therapist competency and client outcomes. Future contributions could focus on refining methods for assessing the impact of DP in supervision groups, including the development of measures for DP group supervisors' competency, and comprehensive assessment of supervisee's skill acquisition and how it may relate to specific in‐session outcomes.

## Data Availability

The data that support the findings of this study are available from the corresponding author upon reasonable request.
